# Can the Bladder Stimulation Technique Solve the Urine Collection Chaos in the Pediatric Emergency Department? A Prospective Comparison with the Traditional Bag Method

**DOI:** 10.3390/children13080999

**Published:** 2026-07-28

**Authors:** Aytaç Göktuğ, İhsan Özdemir, Deniz Karakaya, Şule Demir, Göksel Vatansever, Gülser Esen Besli, Ergin Çiftçi, Deniz Tekin

**Affiliations:** 1 Department of Pediatric Emergency Medicine, Göztepe Prof. Dr. Süleyman Yalçın City Hospital, Istanbul Medeniyet University, 34722 Istanbul, Türkiye; 2 Department of Pediatric Emergency Medicine, Faculty of Medicine, Ankara University, 06620 Ankara, Türkiye; iozdemir@ankara.edu.tr (İ.Ö.); tekind@ankara.edu.tr (D.T.); 3 Department of Pediatric Nephrology, Ankara Etlik City Hospital, 06170 Ankara, Türkiye; 4 Department of Pediatric Emergency Medicine, Faculty of Medicine, Aydın Adnan Menderes University, 09100 Aydın, Türkiye; sule.demir@adu.edu.tr; 5 Department of Pediatrics, Faculty of Medicine, Ankara University, 06620 Ankara, Türkiye; 6 Department of Pediatric Infectious Diseases, Faculty of Medicine, Ankara University, 06620 Ankara, Türkiye

**Keywords:** pediatric emergency department, bladder stimulation technique, bag specimen urine, sample contamination

## Abstract

**Highlights:**

**What are the main findings?**

- The bladder stimulation technique demonstrated the highest success rates in infants aged 3 months and under, weighing less than 6 kg, who remain calm during the procedure.
- Compared to the traditional urine bag method, this technique yields significantly lower contamination rates and faster urine collection times.

**What are the implications of the main findings?**

- This new technique has the potential to replace the traditional urine bag—the most frequently used method in emergency departments—which is often hindered by long waiting times and high contamination rates.
- By providing a faster and more reliable alternative, it may reduce the need for invasive procedures, facilitate the earlier initiation of proper treatment, and prevent families from leaving the hospital before sample collection.

**Abstract:**

**Background/Objectives**: Non-invasive urine collection in the pediatric emergency department (ED) is frequently complicated by prolonged collection times and unacceptably high contamination rates associated with the traditional bag specimen urine (BSU) method. This prospective study aimed to compare the operational efficiency and diagnostic reliability of the bladder stimulation technique (BST)—a highly promising alternative—against the traditional BSU method. **Methods**: The study included 149 infants aged ≤ 6 months (BST group: *n* = 81; BSU group: *n* = 68) requiring urinalysis. We systematically evaluated procedural success rates, time-to-collection metrics, and sample contamination frequencies. Furthermore, we assessed the influence of patient age, weight, behavioral state, and sex on BST efficacy. **Results**: The BST demonstrated superior clinical performance, significantly reducing the total mean time-to-collection to 21 min, compared to 60 min—often extending up to 4 h—typically required for BSU. Notably, the actual stimulation maneuver required only about 78 s. While approximately half of the BSU samples were contaminated, BST markedly decreased this rate (16% vs. 58%). Factors associated with the highest procedural success included patient age ≤ 3 months, weight ≤ 6000 g, and remaining calm during the procedure. Infant sex did not significantly affect success rates. **Conclusions**: Functioning as both an “operational accelerator” and a “diagnostic firewall,” the BST mitigates the inherent limitations of conventional methods and has the potential to replace the traditional urine bag. By offering a rapid, predictable, and clean alternative, it facilitates the swift initiation of accurate treatment and helps prevent families from leaving the ED before sample collection.

## 1. Introduction

Urinalysis and urine cultures are routinely requested as essential components of clinical evaluation in pediatric emergency departments (PEDs). The clinical utility of these tests extends far beyond suspected urinary tract infections (UTIs); they are frequently indicated for a wide spectrum of presentations, including vomiting, dehydration, metabolic disease screening, lower abdominal pain, and suspected urinary tract anomalies. However, obtaining an appropriate and contamination-free urine sample from pre-toilet-trained children poses a significant challenge in daily clinical practice [1,2]. This challenge not only delays the diagnostic process but also leads to prolonged waiting times for families in overcrowded emergency settings, occasionally causing parents to leave the facility before a sample is collected [2,3].

Although gold-standard techniques, such as urethral catheterization and suprapubic aspiration, offer high diagnostic accuracy, their invasive nature, associated procedural pain, and potential complications often provoke substantial resistance from patients and their families [3,4]. Conversely, non-invasive bag specimen urine (BSU) collection is widely preferred due to its ease of application. However, the BSU method is heavily hindered by prolonged waiting times, lower procedural success rates, and an exceedingly high risk of specimen contamination [3,5,6]. Consequently, the search for alternative non-invasive methods remains highly relevant, particularly when clinicians seek to avoid invasive interventions.

In recent years, the bladder stimulation technique (BST), which aims to induce micturition through suprapubic percussion and lumbosacral massage, has gained significant attention [4,6,7]. This technique is based on a spinal reflex arc localized at the S2–S4 levels, where mechanical stretching of the dilated bladder wall stimulates afferent fibers in the spinal cord, which in turn triggers detrusor muscle contraction and internal sphincter relaxation, allowing urine to pass. In continent individuals, the micturition reflex is voluntarily inhibited and modulated by the cerebral cortex [7]. However, in infants under two years of age, this cortical inhibition is not yet mature. Consequently, voiding is triggered automatically in response to increased intra-abdominal pressure or direct bladder stimulation [8].

Initially described by Herreros et al., this non-invasive method is notable for its remarkable success rates, particularly in neonates under 30 days of age [7]. Subsequent investigators, such as Labrosse and Kaufman, evaluated the technique using various protocols to assess its success, procedural duration, and contamination rates [4,6], while Tran et al. demonstrated its feasibility in overcrowded emergency environments [8]. Recently, Ravichandran et al. examined the nurse-led applicability of the BST in a busy PED setting and reported high parental and healthcare provider satisfaction, along with successful integration into routine clinical workflows [9]. Collectively, these studies suggest that the BST offers a highly tolerable, practical, and satisfying alternative to invasive sampling. Nevertheless, further robust data are required to evaluate its performance regarding contamination rates and procedural duration under real-world emergency conditions.

To address this gap in the literature, we utilized a prospective, quasi-randomized design. The primary objective was to evaluate the success rate of obtaining clean-catch midstream urine samples using bladder stimulation maneuvers in infants aged ≤6 months and to identify the clinical factors influencing this success. The secondary objective was to compare this technique with the conventional BSU method in terms of specimen contamination rates and total emergency department waiting times.

## 2. Materials and Methods

### 2.1. Study Design and Ethical Approval

This study was designed as a prospective, quasi-randomized clinical trial conducted in a high-volume tertiary PED. Ethical approval for the study protocol was granted by the Ankara University Faculty of Medicine Clinical Research Ethics Committee (Date: 14 August 2025; Decision Number: 107-654-25). Written informed consent was obtained from the parents or legal guardians of all infants prior to enrollment. This study is registered at ClinicalTrials.gov (Identifier: [NCT07654725]).

### 2.2. Participant Selection

Infants aged 6 months or younger who presented to the PED and required a urine culture for any clinical indication, based on the clinical judgment of the evaluating physician, were assessed for eligibility. Patients were excluded from the study if they presented with a toxic appearance, poor general condition, hemodynamic instability, clinical scenarios requiring immediate emergency sterile urine collection, inadequate oral intake or clinical signs of dehydration, or concomitant anatomical abnormalities affecting the maneuver site or positioning (e.g., developmental dysplasia of the hip or spina bifida).

### 2.3. Group Allocation and Procedural Workflow

Patient allocation followed a quasi-randomized, convenience sampling approach based on the on-site availability of the principal investigator. To ensure absolute methodological consistency and eliminate operator-dependent variability, the specific suprapubic and paravertebral massage maneuvers for all patients in the BST group were performed exclusively by this single investigator. When the principal investigator was unavailable, patients were assigned to the control group and underwent the standard BSU method. Infants whose parents declined to participate in the study were excluded.

To ensure procedural standardization, the BST consistently involved the active participation of a coordinated three-member team. During the stimulation maneuvers, a second team member held the infant in a vertically suspended position under the axillae. Concurrently, a third team member was responsible solely for holding a sterile container and capturing the clean-catch urine sample. To optimize technical success within this workflow, a sex-specific positioning approach was utilized: female infants were maintained in a hip flexion posture, while male infants’ legs were allowed to dangle freely in a neutral position.

### 2.4. Study Variables and Covariates

In addition to baseline demographic characteristics, the primary and secondary study endpoints evaluated in both groups included procedural success rates, exact time to urine collection, and specimen contamination rates.

To evaluate the specific clinical predictors of procedural success, stratified subgroup analyses based on age (categorized as under 1 month, 1 to 3 months, and over 3 months) and sex (male versus female) were performed across both the BST and BSU groups. Conversely, the specific impacts of body weight (under 4000 g, 4000 to 6000 g, and over 6000 g) and behavioral status during the procedure (classified as minimal/no crying versus vigorous crying) on technique efficacy were investigated exclusively within the BST group.

### 2.5. Technical Procedure

Prior to the initiation of the study, all participating investigators received a standardized 10-min instructional training session on an infant manikin to ensure technical uniformity. As described by Herreros et al. [7], parents were requested to feed their children prior to the BST, and the stimulation maneuver was applied 20 min after feeding. To ensure standardization, parents of the infants in the BSU group were also requested to feed their children before the urine bag was attached. Following standard genital cleansing, patients in the control group were fitted with sterile urine bags (standard institutional supply, Istanbul, Türkiye) immediately after feeding, without any waiting period (BSU group). Following the application of the sterile perineal bag, the infants were continuously monitored by their parents. To minimize prolonged skin contact and subsequent contamination, the bags were visually checked for urination at frequent intervals (approximately every 10 to 15 min). The urine sample was collected immediately upon the detection of spontaneous micturition. Thus, the collection time recorded for this group reflects the actual duration from bag application to spontaneous voiding, rather than a predetermined systematic waiting period. For patients in the experimental group (BST group), a mandatory 20-min waiting period was strictly observed post-feeding. The stimulation protocol was then initiated exactly 20 min post-feeding by the designated three-member team, utilizing the sex-specific positioning outlined above.

**Figure 1:**
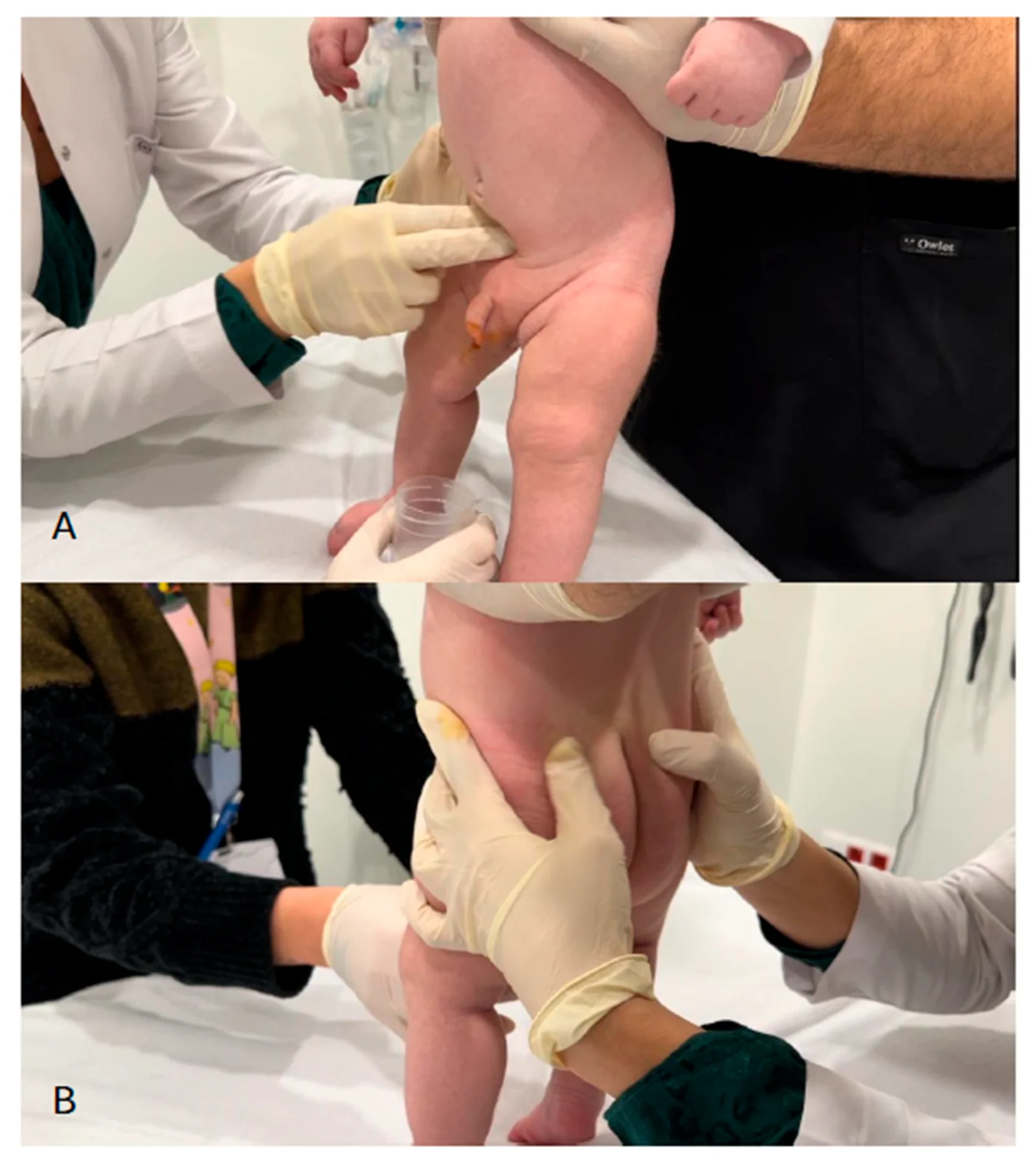
Male infant positioning for urine collection using the bladder stimulation technique. (**A**) Tapping in the suprapubic area. (**B**) Massage to the lower back.

During the procedure, the principal investigator applied rhythmic tapping to the suprapubic region using the second and third fingers at a rate of 100 beats per minute for 30 s. This was immediately followed by a 30-s lumbar paravertebral massage performed with both thumbs in circular motions. These two maneuvers were repeated successively until micturition commenced or the maximum designated duration of 5 min was reached, with the urine sample being captured concurrently. The bladder stimulation maneuver performed on a male infant is illustrated in Figure 1.

### 2.6. Operational Definitions

Procedural success was defined as the collection of 1 mL or more of urine within a 5-min stimulation period for the BST group, or upon successful collection following bag attachment for the BSU group [4]. The time-to-urine collection for the BST group included the mandatory 20-min post-feeding wait plus the exact duration of the stimulation maneuver, whereas for the BSU group, it was calculated as the total elapsed time from bag attachment to successful collection.

Regarding microbiological outcomes, a UTI was defined as the uniform isolation of a single uropathogen with a colony count of 10^5^ CFU/mL or greater. ‘Contamination’ was characterized by the presence of mixed bacterial growth (two or more distinct organisms) regardless of the total colony count. Results yielding a single organism strictly below the diagnostic threshold (fewer than 10^5^ CFU/mL) were classified as ‘no significant growth’, while the complete absence of bacterial proliferation on the culture was defined as ‘no growth’ [2].

Consistent with the literature, sample contamination was practically defined as mixed bacterial growth [2,10] Due to the cross-sectional design of the study and ethical considerations regarding the avoidance of unnecessary invasive procedures in clinically stable infants, a concurrent gold-standard control group (e.g., urethral catheterization or suprapubic aspiration) was not established. 

### 2.7. Participants and Sample Size Determination

The sample size was determined based on the success rates of the BST and standard BSU methods reported in the literature [11]. Assuming a 95% confidence level (1 – *α*) and 80% test power (1 – *β*), the minimum required sample size of 124 participants (BST = 62, BSU = 62) was calculated to provide an effect size of w = 0.345.

### 2.8. Statistical Analysis

Statistical analyses were performed using the Statistical Package for the Social Sciences (SPSS), version 24.0 (IBM Corp., Armonk, NY, USA). The normality of the continuous variables was evaluated using the Shapiro-Wilk test. For the comparison of continuous variables between the groups, the independent samples t-test was utilized for normally distributed data, whereas the Mann-Whitney U test was applied for non-normally distributed data. Categorical variables were compared using the chi-square test. Statistical significance was set at a *p*-value of less than 0.05. Multivariate analysis was performed to identify independent predictors of BST success (age, weight, behavioral state, and sex).

### 2.9. Use of Generative AI and AI-Assisted Technologies in the Writing Process

During the preparation of this manuscript, the authors used Google Gemini Version 1.5 Pro (Google LLC, Mountain View, CA, USA; 2026) to improve readability, refine the English language, and assist with structural formatting. After using this tool, the authors rigorously reviewed, edited, and validated all content, and take full responsibility for the accuracy, integrity, and originality of the final publication.

## 3. Results

### 3.1. Study Population and Baseline Characteristics

A total of 149 infants aged 0–6 months were included in the study. The indication for obtaining a urine culture was established by the attending pediatric emergency physicians based on their clinical evaluation of the patients’ presenting complaints. Following this expert clinical judgment, the most common presenting symptom prompting a urine culture was fever (*n* = 62, 41.6%). Other presenting complaints included jaundice (*n* = 27, 18.1%), irritability (*n* = 25, 16.8%), decreased feeding (*n* = 15, 10.1%), vomiting (*n* = 13, 8.7%), and malodorous urine (*n* = 7, 4.7). Of the 70 male infants included in the study, only 3 were circumcised (1 in the BSU group, 2 in the BST group); thus, the male cohort was overwhelmingly (95.7%) uncircumcised. As detailed in the Section 2, the included patients were assigned to either the bladder stimulation technique (BST) group (*n* = 81) or the conventional bag specimen urine (BSU) group (*n* = 68). The demographic and clinical characteristics of the study groups are summarized in Table 1. The median age and monthly distribution rates were comparable between the two groups. Males accounted for 38.3% and 57.4% of the BST and BSU groups, respectively (*p* = 0.020). Due to concurrent defecation and subsequent sample contamination during the procedure, urine specimens from four patients (8.0%) in the BST group and two patients (4.0%) in the BSU group could not be sent for laboratory analysis.

### 3.2. Procedural Efficacy and Time Metrics

The procedural success rate was 59.3% (48/81) in the BST group and 79.4% (54/68) in the BSU group, revealing a statistically significant difference (*p* = 0.008). However, among the patients with successful urine collection, the median duration from the completion of feeding to obtaining the sample was 21.3 min (IQR: 20.4–22.4; range: 20.1–24.7) in the BST group (*n* = 48), which was significantly shorter than the 60.0 min (IQR: 30.0–90.0; range: 5–235) observed in the BSU group (*n* = 54) (*p* < 0.001).

Within the BST group, the median time to successful micturition during the active stimulation maneuver itself was 78 s (range: 6–282 s). Although vigorous crying was observed in 53.1% of the infants during the procedure, it did not necessitate the discontinuation of the maneuver in any patient.

### 3.3. Microbiological Outcomes

Regarding microbiological outcomes, a statistically significant difference was observed between the two collection methods (*p* < 0.001). While the rate of UTI was comparable between the groups (4.5% in the BST group vs. 5.8% in the BSU group), the contamination rates were markedly different. The contamination rate was significantly lower in the BST group (18.2%) than in the BSU group (55.8%). Correspondingly, cultures yielding no growth were obtained in 68.2% of the BST group, whereas this rate was only 26.9% in the BSU group.

A detailed subgroup analysis based on sex and collection method revealed distinct contamination patterns. In the BSU group (*n* = 68; 39 males, 29 females), contamination rates were notably high across both sexes, particularly among males, occurring in 48.7% of males (19/39) and 34.5% of females (10/29). Conversely, in the BST group (*n* = 81; 31 males, 50 females), not only was the overall contamination significantly reduced, but the rates were also equalized between sexes, observed in only 9.7% of males (3/31) and 10.0% of females (5/50).

### 3.4. Predictors of BST Success

The predictors of successful urine collection using the BST are detailed in Table 2. Regarding age distribution within the BST group, the procedural success rate was 84.0% in neonates younger than 1 month, which significantly declined to 64.7% in infants aged 1 to 3 months, and further to 22.7% in those older than 3 months (*p* < 0.001). A similar inverse relationship was observed concerning body weight; success rates were 84.0% for infants weighing under 4000 g, 60.0% for those weighing 4000 to 6000 g, and 28.6% for those over 6000 g (*p* = 0.001).

Multivariate logistic regression analysis demonstrated that the success of the BST was significantly and independently influenced by age, body weight, and the infant’s behavioral state. Infants aged 3 months or younger were nearly three times more likely to achieve successful urine collection compared to those older than 3 months (OR: 2.90, 95% CI: 1.77–4.59, *p* < 0.001). Similarly, body weight was identified as a strong predictor of the outcome; infants weighing 6000 g or less had a 2.4-fold higher likelihood of procedural success than heavier infants (OR: 2.40, 95% CI: 1.49–3.82, *p* = 0.001). Notably, the behavioral state of the infant during the maneuver also played a critical role, as absent or minimal crying increased the odds of success by 2.4 times (OR: 2.40, 95% CI: 1.26–4.42, *p* = 0.003). In contrast, sex was not identified as a significant predictor of procedural success in the multivariate model (OR: 1.10, 95% CI: 0.63–1.88, *p* = 0.477).

## 4. Discussion

The primary finding of our study is that BST is a highly effective, non-invasive method for urine collection in infants, with its procedural success being strongly and independently predicted by an age of 3 months or younger, a body weight of 6000 g or less, and a calm behavioral state. Beyond identifying these critical predictors, our research distinguishes itself by providing a rigorous head-to-head comparison with the traditionally utilized BSU. Notably, BST demonstrated clear superiority over BSU, resulting in a substantially shorter duration to successful collection and a markedly lower contamination rate. While both methods exhibited a similar capacity for detecting UTI, the combined clinical advantages of superior speed and diagnostic reliability position BST as a much more effective and practical approach for clinical decision-making in time-sensitive pediatric emergency settings.

### 4.1. Clinical Efficacy and Predictors of Success

In the PEDs, obtaining a reliable urine sample from non-toilet-trained children remains one of the most significant diagnostic hurdles. The BST has emerged as a promising alternative, offering high success rates and minimizing contamination. Herreros Fernández et al. observed a high success rate of 86.0% specifically within the neonatal population [7]. Labrosse et al. reported an overall success rate of 49.0% in infants under 6 months of age, with a higher efficacy in those younger than 3 months [4]. Meanwhile, Tran et al. demonstrated that an 89.0% success rate in the neonatal period progressively declined to 29.0% after one year of age, a trend closely mirrored by a weight-dependent reduction from 86.0% in infants weighing under 4 kg to 29.0% in those exceeding 10 kg [8].

Our findings closely align with and expand upon these historical data, demonstrating an overall success rate of 59.3% in infants aged 6 months or younger. The success rate reached 84.0% in neonates under one month but significantly declined to 64.7% in the 1 to 3 months group and dropped to 22.7% in infants older than 3 months. A nearly identical trend was observed regarding weight: an 84.0% success rate in infants under 4000 g dropped to 60.0% in the 4000–6000 g range, and further plummeted to 28.6% in those exceeding 6000 g. Multivariable logistic regression analysis identified age as the most potent independent predictor of procedural success, with infants aged 3 months or younger being nearly three times more likely to achieve successful urine collection (OR: 2.90, 95% CI: 1.77–4.59, *p* < 0.001). Similarly, body weight emerged as a major determinant; infants weighing 6000 g or less demonstrated a 2.4-fold increase in the likelihood of success compared with heavier infants (OR: 2.40, 95% CI: 1.49–3.82, *p* = 0.001).

These age- and weight-dependent declines strongly underscore the neurophysiological basis of this technique, which leverages primitive spinal reflex arcs. While Kaufman et al. characterized this mechanism as an exteroceptive somato-bladder reflex triggered by cutaneous stimulation, it is frequently identified as the Pérez reflex in clinical practice [6]. This spinal reflex, responsible for involuntary bladder emptying in response to specific cutaneous triggers, is most robust in early infancy and gradually diminishes as cortical inhibition and central nervous system maturity develop with age.

### 4.2. The Neurophysiological Role of Behavioral State

A particularly notable and novel finding of our study is the significant influence of the infant’s behavioral state on the success of the BST. In our cohort, infants who remained calm (exhibiting absent or minimal crying) during the procedure were 2.4 times more likely to achieve successful voiding than those who were distressed or exhibited vigorous crying (OR: 2.40, 95% CI: 1.26–4.42, *p* = 0.003).

This strong correlation provides a critical new perspective on the physiological mechanism of the maneuver. Cutaneous stimulation is hypothesized to trigger a parasympathetic-mediated detrusor contraction via the somato-bladder reflex. However, severe distress and vigorous crying induce a systemic sympathetic surge, which likely overrides this delicate parasympathetic reflex, increasing sphincter tone and inhibiting micturition. Therefore, maintaining patient comfort is not merely a matter of procedural elegance, but a neurophysiological prerequisite for success.

Viewed through this physiological lens, the BST offers a distinct advantage over other reflex-triggering techniques, such as the Quick-Wee method. In their landmark trial, Kaufman et al. utilized cutaneous stimulation with gauze soaked in cold saline, noting that crying and mild distress were common occurrences (appropriately classified as expected procedural behaviors rather than adverse events) [6]. While the Quick-Wee method is effective, the sudden application of a cold stimulus inevitably induces thermal discomfort. By avoiding cold-induced irritation, the purely mechanical stimulation of the BST naturally promotes a calmer behavioral state, thereby preventing sympathetic override and optimizing the reflex arc. Furthermore, beyond these physiological benefits, the BST requires no temperature-dependent preparations or additional supplies (e.g., cold saline or ice). This combination of optimal patient comfort and logistical simplicity makes it a highly practical and advantageous alternative for rapid urine collection in a busy pediatric emergency department.

Consequently, we suggest that integrating non-pharmacological soothing interventions, such as the strategic use of pacifiers or the timely administration of oral dextrose, with the BST may further enhance procedural success by maintaining a favorable behavioral state and facilitating optimal technical performance. Future prospective studies are warranted to evaluate the efficacy of combining these specific comfort measures with standardized bladder stimulation protocols.

### 4.3. The Influence of Infant Sex on Procedural Success

In contrast to these age-, weight-, and behavior-related factors, sex did not emerge as a significant predictor of procedural success in our multivariable model, a finding consistent with the existing literature [4,7]. Male infants showed only a marginal, non-significant increase in success probability compared to females (OR: 1.10, 95% CI: 0.63–1.88, *p* = 0.477), suggesting that the BST is equally effective across both sexes and that its physiological reflex mechanism is not sex-dependent. Importantly, although a baseline imbalance in sex distribution was noted between the study groups (*p* = 0.020), these multivariable findings confirm that this initial discrepancy did not act as a confounding factor, thereby reinforcing the overall reliability of our results.

### 4.4. Operational Efficiency: BST vs. Traditional BSU

Beyond identifying the critical predictors of procedural success, our study fundamentally distinguishes itself from the existing literature by providing a comprehensive head-to-head clinical comparison of the widely utilized BSU method. Although BSU remains the most frequently employed technique for non-toilet-trained infants, its practical limitations, including prolonged waiting times and repeated replacements due to leakage or immediate contamination, are well recognized. These operational constraints not only impose a repetitive workload on healthcare staff but also significantly diminish parental satisfaction.

The BST was developed as a dynamic alternative to midstream urine collection. Current literature reports median procedural success times for the BST itself ranging from 45 to 73 s in infants under six months of age [4,7,9,12,13]. In our cohort, the median duration of a successful BST was 78 s, consistent with these established benchmarks. Building on this finding, a major methodological strength of our study is that we calculated the total urine collection time in addition to the isolated procedural duration. While previous research has often focused strictly on the procedural moment, we evaluated the entire clinical workflow. This comprehensive measurement incorporated the mandatory 20-min post-feeding waiting period for the BST group, comparing the total “time-to-collection” directly against the total elapsed time required for successful sample acquisition via the traditional BSU.

Although the BSU method yielded a higher raw procedural success rate than the BST (79.4% vs. 59.3%), this comparison must be interpreted in the context of our pragmatic study design. There is an inherent asymmetry in how procedural success was defined between the two groups. By definition, success in the BSU group meant the eventual collection of a urine sample without strict time limitations, making it a passive process that relies on prolonged waiting periods. In contrast, the BST was evaluated as a rapid, active, and time-bound intervention. Rather than a methodological flaw, we believe this discrepancy accurately reflects the real-world operational nature of these two techniques in a busy PED setting.

Consistent with Kapoor et al., who reported an 86.7% BSU success rate with a median duration of 70 min [12], our BSU group achieved a high success rate, albeit at the expense of prolonged waiting periods. While the median collection time was 60.0 min, the process extended up to 235 min in extreme cases. In stark contrast, the median total workflow duration for the BST was only 21.3 min (range: 20.1–24.7 min) (*p* < 0.001). While a one-hour median waiting time is already a substantial burden in acute care, operational outliers reaching nearly four hours represent severe systemic bottlenecks. Such unpredictable delays are known to increase the risk of patients leaving the PED prematurely (leaving without being seen [LWBS]) [1]. Although our study did not specifically measure LWBS rates, the significant reduction in time-to-collection achieved via BST inherently mitigates the key drivers of parental exhaustion and PED overcrowding. Ultimately, by eliminating the uncertainty of traditional BSU, the BST provides a rapid diagnostic tool that fundamentally aligns with the core philosophy of emergency medicine: delivering swift and definitive care.

### 4.5. Diagnostic Reliability and Contamination

The most significant barrier to accurate UTI diagnosis in un-toilet-trained infants is sample contamination by perineal flora. While the literature reports high contamination rates for the BSU method (45–63%), these rates are significantly lower for the BST (6.8–24%) [1,5,9,14]. Our findings strongly align with these data, demonstrating a contamination rate of 55.8% in the BSU group compared to only 18.2% in the BST group. Although there was a higher proportion of males in our BSU cohort—a demographic generally associated with a higher baseline risk of contamination—this imbalance alone cannot explain the high overall contamination rate observed with the bag method. In our study, BSU contamination was unacceptably high in both sexes (48.7% [19/39] in males and 34.5% [10/29] in females). Conversely, the BST effectively neutralized this issue, markedly reducing and equalizing the contamination rates across both sexes (9.7% for males and 10.0% for females).

This universal improvement achieved by the BST can be explained by stream dynamics. A pivotal study focusing specifically on uncircumcised boys—mirroring the overwhelming majority of our male cohort—demonstrated that contamination is heavily concentrated in the initial urine stream (reported as 51%) due to the flushing of the preputial flora, whereas midstream samples remain significantly cleaner (16%) [15]. It is highly probable that a similar physiological mechanism applies to female infants, where the initial urine stream flushes the dense periurethral and perineal flora. Because the traditional bag method pools the entire micturition, it inherently collects this highly contaminated initial flush. In contrast, during the BST procedure, the brief reaction time required for the practitioner to position the sterile cup invariably allows the first few drops of urine to escape. Consequently, the BST naturally captures a high-quality midstream sample. This mechanical bypass of the initial preputial and perineal bacterial load provides a methodological superiority that protects both male and female infants from sample contamination, thereby reducing false-positive cultures, unwarranted antibiotic use, and unnecessary hospital admissions

Conversely, the 18.2% contamination rate achieved with BST closely approximated the diagnostic reliability of invasive procedures. Because of its high diagnostic yield, a significant portion of the existing literature has historically focused on comparing the BST primarily with these invasive techniques. Consequently, direct head-to-head clinical comparisons with the noninvasive standard, the BSU method, remain remarkably scarce and are often strictly restricted to the neonatal period. For example, Kapoor et al. noted that while BST was faster than BSU in neonates (55 s vs. 70 min), the overall success rates were similar (88% vs. 86%) [12]. Furthermore, a recent 2024 neonatal study reported a higher success rate within a three-minute window for BST compared to BSU (62.5% vs. 28%) [11]. However, neither of these head-to-head trials evaluated or compared sample contamination rates between the two methods, leaving a major blind spot in their clinical utility. Our research uniquely bridges these critical gaps by expanding the investigated population to six months of age, providing a neurophysiological context for the success rates, and demonstrating a stark, definitive contrast in sample contamination. Ultimately, our findings validate that BST serves as both a “diagnostic safeguard” and an “operational accelerator,” positioning it as a highly effective, practice-changing intervention in acute pediatric care.

Our findings offer substantial contributions to the existing pediatric emergency literature by providing clear, data-driven clinical implications. While previous studies have evaluated various non-invasive techniques, our results strongly suggest that the BST should transition from being merely an alternative to becoming the preferred, first-line non-invasive urine collection method in the PED for appropriately selected patients.

Regarding patient selection, we recommend prioritizing the BST for infants especially under 3 months of age who are hemodynamically stable, show no clinical signs of dehydration, lack any physical contraindications for the required positioning, and have cooperative parents. Conversely, in critically ill infants or those requiring an emergent UTI diagnosis and immediate initiation of treatment, invasive methods should not be delayed.

Crucially, the BST is universally applicable for all infants meeting these clinical criteria, as it effectively circumvents anatomical and methodological vulnerabilities to yield equal procedural success and drastically reduce contamination in both sexes, thereby justifying its implementation as the standard non-invasive protocol to prevent false-positive UTI diagnoses, unnecessary antibiotic prescriptions, and avoidable catheterizations, while significantly shortening emergency department waiting times.

### 4.6. Limitations

Our study has several limitations that should be considered when interpreting the findings. First, although the study was conducted in a high-volume PED, the single-center design may limit the generalizability of our results to different clinical settings. Second, to ensure strict procedural standardization, all bladder stimulation maneuvers were performed by a single, trained investigator. While this approach maximized technical consistency and eliminated inter-operator variability, it necessitated a convenience sampling approach dependent solely on the investigator’s availability, thereby precluding formal randomization. Future multicenter studies involving a diverse range of practitioners are warranted to validate these findings across broader populations.

Additionally, while we qualitatively categorized infant behavior during the maneuver, patient comfort and distress were not assessed using validated pain scoring tools or continuous vital sign monitoring, which would have provided a more objective measure of the physiological impact of the procedure. Furthermore, the lack of a universal consensus regarding the definition of urinary sample contamination in the pediatric literature may lead to diagnostic ambiguity and inconsistent definitions in clinical practice. Finally, our study design did not include a protocol to cross-validate the obtained samples—particularly those categorized as contaminated—against a gold-standard invasive method, such as suprapubic aspiration or urethral catheterization. Although patients with contaminated results in routine clinical practice often undergo subsequent invasive sampling for a definitive diagnosis, our cross-sectional study design focused solely on the initial emergency department presentation; thus, longitudinal follow-up data to retrospectively cross-validate these contaminated cases were not collected. The significance of relying solely on mixed bacterial growth to define contamination is that while it is a widely accepted practical standard for evaluating non-invasive samples, it inherently lacks the diagnostic absolute of a sterile comparator. Consequently, this definition may fail to identify low-colony-count single-organism contaminations or differentiate them from true asymptomatic bacteriuria. This implies that the true contamination rate for both non-invasive methods evaluated in this study could potentially be underestimated. Despite these limitations, our results provide a robust and highly practical framework for the implementation of the BST in acute pediatric care.

## 5. Conclusions

The BST is a highly effective and reliable alternative to traditional urine collection methods in acute pediatric care. The technique yields its highest procedural success rates in infants aged 3 months or younger, those weighing 6000 g or less, and those who remain calm during the maneuver. By significantly reducing the time-to-collection and inherently capturing a cleaner midstream sample, it ensures markedly lower contamination rates across both sexes compared to the traditional BSU. The BST therefore serves as both an ‘operational accelerator’ and a ‘diagnostic firewall.’ These findings demonstrate that bladder stimulation provides a rapid, predictable, and clean sampling method that directly addresses the inherent limitations of conventional, non-invasive approaches. Ultimately, by offering a superior balance of diagnostic efficiency and clinical reliability, the BST stands as a practice-changing intervention that fundamentally aligns with the core philosophy of emergency medicine: delivering swift and definitive care.

## Figures and Tables

**Table 1:** Comparison of demographic and clinical characteristics between the BST and BSU groups.

	BST Group (N = 81)	BSU Group (N = 68)	*p*
Age ^a^, month	1.8 (0.9–3.2)	1.2 (0.6–3.0)	0.576
Age group ^b^			0.810
<1 month	25 (30.9)	23 (33.8)	
1–3 months	34 (42.0)	25 (36.8)	
>3 months	22 (27.2)	20 (29.4)	
Sex, male ^b^	31 (38.3)	39 (57.4)	0.020 *
Success of the method ^b^	48 (59.3)	54 (79.4)	0.008 **
Success by age group <1 month	21 (84.0)	14 (60.9)	
1–3 months	22 (64.7)	23 (92.0)	
>3 months	5 (22.7)	17 (85.0)	
Urine collection time ^a,c^, min	21.3 (20.4–22.4)	60 (30.0–90.0)	<0.001 **
Culture result ^b^	44 (54.0)	52 (77.0)	<0.001 **
Significant growth	2 (4.5)	3 (5.8)	
Insignificant growth	4 (9.1)	6(11.5)	
Contamination	8 (18.2)	29 (55.8)	
No growth	30 (68.2)	14 (26.9)	

^a^: Median (25th–75th percentiles), ^b^: number (%), ^c^: The median urine collection times were calculated exclusively based on the successful attempts for each group (*n* = 48 out of 81 for BST; *n* = 54 out of 68 for BSU), excluding cases where the methods failed. * *p* < 0.05, ** *p* < 0.01. BST: Bladder Stimulation Technique, BSU: Bag Sample Urine. **Note:** The total number of culture results does not match the total number of successful procedures because four samples in the BST group and two samples in the BSU group could not be sent for culture analysis due to fecal contamination.

**Table 2:** Factors affecting the success of the bladder stimulation technique.

Outcomes	Successful (N = 48) *n* (%)	Unsuccessful (N = 33) *n* (%)	OR (95% CI)	*p*
Sex				
Male	19 (61.3)	12 (38.7)	1.10 (0.63–1.88)	0.477
Female	29 (58.0)	21 (42.0)	1.0 (Ref)	
Age				
<1 month	21 (84.0)	4 (16.0)		
1–3 month	22 (64.7)	12 (35.3)		
>3 months	5 (22.7)	17 (77.3)	1.0 (Ref)	
Total < 3 months	43 (72.9)	16 (27.1)	2.90 (1.77–4.59)	<0.001 **
Weight				
<4000 g	21 (84.0)	4 (16.0)		
4000–6000 g	21 (60.0)	14 (40.0)		
>6000 g	6 (28.6)	15 (71.4)	1.0 (Ref)	
Total ≤ 6000 g	42 (70.0)	18 (30.0)	2.40 (1.49–3.82)	0.001 **
Crying, *n* (%)				
Absent/minimal	29 (76.3)	9 (23.7)	2.40 (1.26–4.42)	0.003 **
Vigorous	19 (44.2)	24 (55.8)	1.0 (Ref)	

** *p* < 0.01, OR: Odds Ratio, CI: Confidence Interval, Ref: Reference. Note: Data are presented as *n* (row %).

## Data Availability

The data presented in this study are available on request from the corresponding author. The data are not publicly available due to privacy and ethical restrictions regarding pediatric patient information.

## Abbreviations

The following abbreviations are used in this manuscript:

BST	Bladder Stimulation Technique
BSU	Bag Specimen Urine
PED	Pediatric Emergency Department
UTI	Urinary Tract Infection
